# Supplementary material for: Study protocol for resolution of organ injury in acute pancreatitis (RESORP): an observational prospective cohort study
Source: BMJ Open. 2020 Dec 7;10(12):e040200. doi: 10.1136/bmjopen-2020-040200 (PMC7722833; doi:10.1136/bmjopen-2020-040200)
Supplement: Author's manuscript [file bmjopen-2020-040200.draft_revisions.pdf]

BMJ Open

BMJ Open is committed to open peer review. As part of this commitment we make the peer review history of every article we publish publicly available.

When an article is published we post the peer reviewers' comments and the authors' responses online. We also post the versions of the paper that were used during peer review. These are the versions that the peer review comments apply to.

The versions of the paper that follow are the versions that were submitted during the peer review process. They are not the versions of record or the final published versions. They should not be cited or distributed as the published version of this manuscript.

BMJ Open is an open access journal and the full, final, typeset and author-corrected version of record of the manuscript is available on our site with no access controls, subscription charges or pay-per-view fees (http://bmjopen.bmj.com).

If you have any questions on BMJ Open's open peer review process please email info.bmjopen@bmj.com

BMJ Open

Study Protocol for RESORP – Resolution of Organ Injury in Acute Pancreatitis – an observational prospective cohort study.

Journal:	*BMJ Open*
Manuscript ID	bmjopen-2020-040200
Article Type:	Protocol
Date Submitted by the Author:	08-May-2020
Complete List of Authors:	Sherif, Ahmed; The University of Edinburgh, Clinical Surgery<br>McFadyen, Rory; The University of Edinburgh,<br>Boyd, Julia; Edinburgh Clinical Trials Unit<br>Ventre, Chiara; The University of Edinburgh,<br>Glenwright, Margaret; NHS Lothian<br>Walker, Kim; NHS Lothian<br>Zheng, Xiaozhong; University of Edinburgh, Centre for Inflammation Research<br>White, Audrey; NHS Lothian<br>McFadyen, Laura; NHS Lothian<br>Connon, Emma; NHS Lothian<br>Damaskos, Dimitrios<br>Steven, Michelle; University of Edinburgh, Edinburgh Clinical Trials Unit<br>Wackett, Anthony<br>Thomson, Euan; NHS Lothian<br>Cameron, David; NHS Lothian<br>Semple, Scott<br>Morris, David<br>Clark-Stewart, Saskia; NHS Lothian<br>Graham, Catriona<br>Mole, Damian; University of Edinburgh MRC Centre for Inflammation Research, ; The University of Edinburgh, Clinical Surgery
Keywords:	Pancreatic disease < GASTROENTEROLOGY, Adult intensive & critical care < ANAESTHETICS, General diabetes < DIABETES & ENDOCRINOLOGY

SCHOLARONE™
Manuscripts

BMJ

I, the Submitting Author has the right to grant and does grant on behalf of all authors of the Work (as defined in the below author licence), an exclusive licence and/or a non-exclusive licence for contributions from authors who are: i) UK Crown employees; ii) where BMJ has agreed a CC-BY licence shall apply, and/or iii) in accordance with the terms applicable for US Federal Government officers or employees acting as part of their official duties; on a worldwide, perpetual, irrevocable, royalty-free basis to BMJ Publishing Group Ltd ("BMJ") its licensees and where the relevant Journal is co-owned by BMJ to the co-owners of the Journal, to publish the Work in this journal and any other BMJ products and to exploit all rights, as set out in our licence.

The Submitting Author accepts and understands that any supply made under these terms is made by BMJ to the Submitting Author unless you are acting as an employee on behalf of your employer or a postgraduate student of an affiliated institution which is paying any applicable article publishing charge ("APC") for Open Access articles. Where the Submitting Author wishes to make the Work available on an Open Access basis (and intends to pay the relevant APC), the terms of reuse of such Open Access shall be governed by a Creative Commons licence – details of these licences and which Creative Commons licence will apply to this Work are set out in our licence referred to above.

Other than as permitted in any relevant BMJ Author's Self Archiving Policies, I confirm this Work has not been accepted for publication elsewhere, is not being considered for publication elsewhere and does not duplicate material already published. I confirm all authors consent to publication of this Work and authorise the granting of this licence.

For peer review only

Study Protocol for RESORP – Resolution of Organ Injury in Acute Pancreatitis – an observational prospective cohort study

Authors

Ahmed E Sherif[1*], Rory McFadyen[1*], Julia Boyd[2*], Chiara Ventre[1], Margaret Glenwright[3], Kim Walker[3], Xiaozhong Zheng[4], Audrey White[3], Laura McFadyen[3], Emma Connon[3], Dimitrios Damaskos[1], Michelle Steven[2], Anthony Wackett[2], Euan Thomson[5], David C Cameron[5], Scott Semple[6], David Morris[4], Saskia Clark-Stewart[1], Catriona Graham[7], and Damian J Mole[1,4**] on behalf of the RESORP research team

*indicates joint 1st authors

** indicates corresponding author

Affiliations

[1] Clinical Surgery, University of Edinburgh, Royal Infirmary of Edinburgh, 51 Little France Crescent, Edinburgh, EH16 4TJ, UK

[2] Edinburgh Clinical Trials Unit, Nine Bioquarter, Edinburgh EH16 4UX, UK

[3] Clinical Research Facility, NHS Lothian, Edinburgh, EH16 4TJ, UK

[4]Centre for Inflammation Research, University of Edinburgh, Queen's Medical Research Institute, 47 Little France Crescent, Edinburgh, EH16 4TJ, UK

[5] Department of Anaesthesia, NHS Lothian, Edinburgh, EH16 4TJ, UK

[6]Centre for Cardiovascular Science, University of Edinburgh, Queen's Medical Research Institute, Edinburgh BioQuarter, 47 Little France Crescent, Edinburgh, EH16 4TJ, UK

[7]Epidemiology and Statistics Core, Edinburgh Clinical Research Facility, University of Edinburgh, Western General Hospital, Crewe Road South, Edinburgh EH4 2XU

Correspondence to: Prof Damian J Mole; damian.mole@ed.ac.uk, +44 (131) 242 9100

For peer review only - http://bmjopen.bmj.com/site/about/guidelines.xhtml

ABSTRACT

Introduction: Survivors of acute pancreatitis (AP) have shorter overall survival and increased incidence of new-onset cardiovascular, respiratory, liver and renal disease, diabetes mellitus and cancer compared to the general population, but the mechanisms that explain this are yet to be elucidated. Our aim is to characterise the precise nature and extent of organ dysfunction following an episode of AP.

Methods and Analysis

This is an observational prospective cohort study in a single centre comprising an acute University hospital and clinical research facility. Participants will be adult patient admitted with AP. Participants will undergo assessment at recruitment, 3 months and 3 years. At each time point, multiple biochemical and/or physiological assessments to measure cardiovascular, respiratory, liver, renal and cognitive function, diabetes mellitus, and quality of life. Recruitment was from 30th November 2017 to 31st May 2020; last follow-up measurements is due 31st May 2023. The primary outcome measure is the incidence of new-onset type 3c diabetes mellitus during follow-up. Secondary outcome measures include: quality of life analyses (SF-36, GIQLI); Montreal cognitive assessment; organ system physiological performance; multiomics predictors of AP severity, detection of premature cellular senescence. In a nested cohort within the main cohort, individuals may also consent to multiparameter MRI scan, echocardiography, pulmonary function testing, cardiopulmonary exercise testing and pulse-wave analysis.

Ethics and dissemination: This study has received the following approvals: UK IRAS Number 178615; South-east Scotland Research Ethics Committee number 16/SS/0065. Results will be made available to AP survivors, caregivers, funders and other researchers. Publications will be open-access.

Trial registration: ClinicalTrials.gov Identifier (NCT03342716) and ISRCTN 50581876; Pre-results

Strengths and limitations

Strengths

- Prospective cohort study with molecular mechanistic observations.
- In-depth clinical and physiological annotation of multiomics data.

For peer review only

Limitations

- Drop-out rates in long term prospective AP studies is largely unknown.
- Potential participants with severe AP may lack capacity for consent and recruitment may therefore be slower in this group.

Keywords: long-term outcome; acute pancreatitis; organ failure; post-inflammatory premature senescence

Word Count: 3709

For peer review only

INTRODUCTION

Acute pancreatitis (AP) is an inflammatory disease of the pancreas usually triggered by gallstones or excess alcohol consumption. The incidence of AP ranges from 13 to 45 per 100,00 individuals, is increasing[1], and varies between nations. For example, USA, Finland, Scotland and Germany report higher incidences than England and The Netherlands[2-5]. AP triggers a cascade of inflammatory events causing cell damage and release of damage-associated molecular patterns that trigger inflammation and cytokine release, initiating a systemic inflammatory response resulting in multiple organ dysfunction syndrome (MODS) in 1 in 5 individuals. AP-MODS has a 21% fatality rate during the index episode, and overall in-hospital mortality is 5.2% in Scotland[6 7]. AP is graded into mild, moderate and severe, based on local and systemic determinants of severity as per the most recent AP classifications (Determinant-based Classification[8] and Revised Atlanta Classification[9]). Organ failure in AP most commonly affects the respiratory, renal and cardiovascular systems, but can involve any system[10]. The pattern of organ failure is difficult to predict and a high level of inter-individual variance is seen, regardless of the aetiology. The molecular mechanisms that explain this inter-individual variation in local and systemic inflammatory processes in AP remain unidentified [11]. Multiomics analysis of serial time course blood samples has defined molecular patterns in human AP, called endotypes, but the precise relevance of these endotypes to clinical and therapeutic decision-making is not yet determined[12].

Recently published data from a retrospective analysis of patients with AP has shown that the early development of MODS is associated with an increased mortality rate up to 10 years after the index presentation[13]. This strongly suggests that MODS in acute pancreatitis (AP-MODS) has a persistent and deleterious impact on patients' physiological status, though the exact nature of this pathology remains to be characterised. Existing studies indicate that most deaths are the result of cardiovascular, pulmonary, digestive or malignant disease[14-17]. In addition, the new onset of diabetes (Type 3c DM) or impaired glucose tolerance, peripheral neuropathy and exocrine dysfunction have been documented as sources of morbidity in the years following the index attack of SAP[17 18]. Patients with alcohol-induced pancreatitis may be at particularly increased risk of progression to chronic pancreatitis[15], however the development of long term complications is likely to be influenced by multiple, and as-yet unconfirmed, factors.

For peer review only

The aim of this observational clinical cohort study is to characterise the nature and extent of the pathophysiological impact of severe AP on organ function over the first 3 years after the index AP episode and define the long-term deleterious effect of severe AP. This will be achieved by prospectively measuring organ system function in patients recruited during a hospital admission with AP and at set time intervals thereafter. We will obtain an in-depth assessment of patients' health at presentation, and at 3 months and 36 months after the first episode of AP using markers of organ function and/or disease in the peripheral blood. In a nested cohort within the main study cohort, we will conduct cardiorespiratory evaluation tests (including exercise testing), specialised blood tests of the immune system, tests for precision medicine, and imaging to assess structure and function of key organ systems.

The results of this study will inform the design of future interventions designed to improve the long-term prognosis of patients.

METHODS

Ethical approval

The study will be conducted in accordance with the principles of the International Conference on Harmonisation Tripartite Guideline for Good Clinical Practice (ICH GCP). This study has been reviewed and been given a favourable opinion by the South-East Scotland Research Ethics Committee, under the application numbers: REC Number 16/SS/0065; IRAS Number 178615; and R&D approval Lothian R&D Number 2016/0152.

Trials registration

This study was prospectively registered with www.clinicaltrials.gov NCT03342716 and with ISRCTN 50581876.

Study design

This is an observational prospective nested cohort study. The overall cohort is the population presenting with AP that meets the eligibility criteria. For the nested cohort of the study, a stratified block design is to be used to recruit equivalent numbers of patients with AP-MODS versus AP without MODS.

For peer review only

Setting

Single centre academic hospital setting – Royal Infirmary of Edinburgh, NHS Lothian and the Edinburgh Clinical Research Facility, Royal Infirmary of Edinburgh, UK.

Duration

Recruitment started on 30th November 2017 and is scheduled to end on 31st May 2020. The cohort assessment will comprise of 3 study visits. In-depth assessments of a participant's health at presentation, at 3 months and at 36 months follow-up study visit will be obtained. Additional cardiorespiratory evaluation tests, specialised blood tests of the immune system, tests for precision medicine, and imaging to assess structure and function of key organ systems will be conducted in a nested cohort of participants.

Intervention

This is a prospective study examining patient outcomes and trends in patients' physiological status after an index event. Therefore, no intervention or randomisation is proposed.

Measurements

Primary endpoint

- Overall cohort: to define the incidence of new-onset type 3c diabetes mellitus in patients with AP, compared to the age-matched population of Scotland.
- Nested cohort: to define the baseline change in pancreatic fibrosis index measured by multi-parameter MRI between 3 and 36 months after recruitment.

Secondary endpoints

Secondary end-points in the study will be experimental and observational, and will define explicitly, or be preliminary exploratory studies investigating:

i) Genomic predictors of AP severity and resolution of inflammation.
ii) miRNA signatures of disease severity and resolution.
iii) Metabolomic profiling of AP resolution, and development of local and systemic complications.
iv) Premature cellular senescence as a pathological consequence of AP-MODS.

For peer review only

v) Alteration in immune cell subsets and phenotype as a long-term response to AP-MODS.

vi) Cardiovascular and respiratory dysfunction as a legacy of endothelial injury during AP-MODS.

Summary of clinical data to be collected

All participants in the overall and nested cohorts will have the following data collected:

i) Demographic and Clinical Background Information.

ii) Information regarding the acute presentation.

iii) Organ function assessment at recruitment, 3- and 36-months follow-up study visit:

   a. Overall cohort:

      i. Full peripheral venous blood profiling, including cardiac biomarkers, standard biochemistry profiling, and samples retained for miRNA profiling, cytokines, telomere length, metabolomic profiling, proteomic profiling, transcriptomic profiling, genomic profiling, leukocyte subset analysis by flow cytometry.

      ii. Biochemical markers of organ function in urine and samples retained.

      iii. Pancreatic exocrine function test in stool (faecal elastase).

      iv. Nutritional assessment.

      v. Oral glucose tolerance test at 3- and 36-month follow-up study visit (with the exception of insulin dependent diabetics who will have random blood glucose measured only).

      vi. 12 lead electrocardiogram (ECG), blood pressure.

      vii. Peripheral SpO2.

      viii. Sway balance app non-invasive muscle function tests, 6-minute walk test.

      ix. Self-administered Patient Questionnaire

         1. Gastrointestinal Quality of Life Index (GIQLI).

         2. SF-12 Quality of Life.

         3. Montreal Cognitive Assessment.

   b. Nested cohorts:

For peer review only - http://bmjopen.bmj.com/site/about/guidelines.xhtml

i. Pulse wave analysis (with salbutamol inhaler); transthoracic echocardiography.
ii. Spirometry and gas transfer.
iii. Cardiopulmonary exercise (CPEX) testing.
iv. Multiparameter magnetic resonance imaging (MRI) assessment of liver and pancreas.

Venous blood, urine and stool sampling

Blood samples will be obtained from each study participant at each of the three study visits for organ function and specialist assays. A urine sample and a stool sample will be obtained at each of the 3 visits.

Nutritional assessment

Participants will have their height and weight measured in order to calculate their Body Mass Index (BMI), as well as bioimpedance analysis +/- upper arm anthropometry to assess percentage body fat and lean tissue mass. Participants will also be asked to keep a 24-hour food diary to assess whether their calorie and protein intake is sufficient for their nutritional needs at all 3 study visits.

Oral glucose tolerance test

At the 3- and 36-month follow-up study visits an oral glucose tolerance test will be performed. Participants attending the research site for physiological testing will be asked to attend in a fasted state. On arrival participants will have the baseline blood sample taken (using the same venepuncture as other tests above as appropriate), be given a standard 75g anhydrous glucose in cold water or Polycal ® (113mL) which is more palatable and will be followed by 150mL water (total volume should be 250-300mL). After 120 minutes, a second blood sample will be taken (using the same venepuncture as other tests above as appropriate) to measure blood glucose.

An oral glucose tolerance test will not be performed in participants with insulin dependent diabetes. Instead a random glucose sample will be taken at these visits.

Non-invasive muscle function testing, Sway balance test, 6-minute walk test

Manual Muscle Testing will be performed once per visit to assess muscle strength. This will follow structured protocols used to assess muscle strength in survivors of critical illness[19]. The

For peer review only - http://bmjopen.bmj.com/site/about/guidelines.xhtml

strength of muscles involved in shoulder abduction, elbow flexion, wrist extension, hip flexion, knee extension and ankle dorsiflexion will be graded according to the Medical Research Council (MRC) muscle strength scoring system.

Participants will undergo an assessment of balance and postural stability at each visit. Testing will follow a validated protocol using the SWAY Balance mobile testing system[20 21]. Participants will be asked to hold a mobile device against their chest whilst completing a series of stances: standing with both feet together, standing with one foot behind the other, and standing on each leg in turn[21]. Each stance is held for 10 seconds with the eyes closed. The SWAY Balance application will generate a balance score, based on detection of alterations in position during each stance.

A six-minute walk test will be done, according to standard protocols[22].

Multiparameter magnetic resonance imaging (Liver & Pancreas)

A multiparameter MRI scan will be performed on each visit for the nested cohort to measure lipid, iron load and fibrosis in liver and pancreas according to protocols in development at CRIC (Clinical Research Imaging Centre). The total duration of scan will be approximately 30 minutes.

Cardiovascular Analysis

Nested cohort participants will undergo pulse wave analysis (PWA) at the radial artery using a non-invasive tonometer to measure wave reflection, pulse pressure and derived arterial stiffness with the augmentation index at 75 bpm and the administration of two puffs of inhaled salbutamol (200 micrograms each puff) via a spacer device. Transthoracic echocardiography will be performed to measure longitudinal shortening of the myocardium.

Pulmonary Function Testing and Gas Transfer

Spirometry will be undertaken in accordance with guidance from the British Thoracic Society for the nested cohort group[23]. All measurement will be made with participants seated. FEV1 and FVC will be recorded as the best of 3 consistent readings, and compared to the predicted normal values for the participant's height, age, gender and ethnicity. Flow volume measurements will also be obtained. Lung volumes will be measured using the steady state method[23]. Gas Transfer/Diffusion Capacity will be measured using the single breath carbon monoxide transfer factor method[23].

For peer review only

252 *Cardiopulmonary Exercise Testing (CPEX)*

CPEX testing will be carried out on day 2 of patient assessment at all visits for nested cohort participants. Participants will be asked not to smoke for 8 hours prior to the test, and to abstain from other strenuous exercise on the same day.

Supervised CPEX testing will be conducted following a standardised incremental protocol on a magnetically braked cycle ergometer. Work-load ramp will be determined using a standard formula[24]. Testing will be continued until symptomatic limitation, volitional termination, failure to maintain required cadence or if other early termination conditions are met[25]. Breath by breath O_2 and CO_2 analysis will be performed using a fully calibrated metabolic cart. 12-lead ECG and O_2 saturations will be monitored continuously in the pre-test, test and recovery phase. Non-invasive blood pressure will be recorded at rest and at 3-minute intervals during test and recovery phase. The anaerobic threshold (AT) will be independently determined by two assessors using the V-slope method.

Recruitment

All adult patients that meet the inclusion criteria at the participating sites will be recruited where possible. Patients admitted with an elevated serum amylase will be identified and members of the clinical research team will screen these patients for a diagnosis of AP using the *TRAKCare®* patient management system (InterSystems, Massachusetts, USA). Potential participants will be approached by a member of the direct care team to confirm if they are willing to discuss the research with a member of the research team. If the individual agrees, they will be contacted by a member of the research team during their inpatient stay. If potential participants are discharged before they can be approached, they will not be included in the study

Inclusion criteria

The following inclusion criteria will apply to all patients:

i) Confirmed clinical or radiological diagnosis of acute pancreatitis with an elevated serum amylase greater than 300 U/L.

ii) For the potential clinical diagnosis of acute pancreatitis an appropriate clinical history based on compatible clinical features, will be required (i.e. abdominal pain, nausea and/or vomiting), supported by the finding of elevated serum

For peer review only - http://bmjopen.bmj.com/site/about/guidelines.xhtml

amylase greater than 300 U/L. For the radiological diagnosis, if applicable, computerised tomography (CT) and/or ultrasound scan (USS) evidence of acute pancreatitis will be accepted.

Exclusion criteria

i) Patients under 16 years of age.
ii) Prisoners.
iii) Any patient that lacks capacity to consent.

The additional two exclusions below apply only to those patients being considered for the nested cohort study:

iv) Patients not able to undergo MRI scanning for technical reasons will be excluded (e.g. those with cochlear implants, implanted pacemaker)
v) Patients with a known allergy to salbutamol

Co-enrolment to other research studies, including drug, interventional and long-term follow-up studies, will be permitted if this has been agreed and documented by the Chief Investigators of co-enrolling studies.

Statistical analysis plan

Full details of the statistical analysis will be documented in the statistical analysis plan developed prior to any analysis. Any deviations from this will be documented. We will present the data descriptively, where data is categorical numbers and percentages will be presented and where data is continuous number, mean, standard deviation, median, 25th centile, 75th centile, min and max will be presented. Annual incidence of type 3c diabetes will be given as a percentage with an accompanying 95% confidence interval. For the nested cohort we will present the change in fibrosis index over time graphically with accompanying descriptive statistics.

Sample size calculation

Overall sample size calculation has been performed to illustrate how well we will be able to estimate the annual incidence of type 3c diabetes in patients with acute pancreatitis with

For peer review only

varying sample sizes[26 27]. With a sample of 323 we would have 80% power to be able to show a difference from the incidence of diabetes mellitus in the general population (6%) if we observed an incidence in the cohort of 10% or greater. Moreover, this sample size will support the evaluation of the secondary endpoints, allowing us to adjust for any confounding factors.

Nested cohort sample size has been calculated to detect the difference in the 3-months to 36-month change in pancreatic fibrosis index between those with AP with MODS and those with AP without MODS based on Banerjee et al. estimates of fibrosis index using multiparameter MRI[28]. Based on a sample size of 20 participants per group, using a two-sided two-sample test with a 5% level of significance and 80% power, we would be able to detect an effect size of 0.909 (where the effect size is the absolute difference in means divided by common standard deviation). To allow for potential dropouts in this part of the study (death, withdrawal) a minimum sample size of 25 individuals will be recruited for the nested cohort.

Data management

Data will be collected either directly onto an electronic case report form on REDCap (Research Electronic Data Capture) tools hosted at University of Edinburgh[29 30] or onto paper data collection sheets which will be entered onto the electronic form at site. All participants will be allocated a unique study number which will be used in all electronic and paper data collection tools. The study link-anonymised data will be stored on secure password-protected, regularly backed-up, computerised storage in accordance to secure data protection principles of the University of Edinburgh/CRF at study sites.

Personal contact details and the CHI (Community Health Index) number are required for follow-up and tracking electronic health records, and will initially be kept on paper at participating sites in a secure storage area with restricted access. These details will ultimately be stored on an encrypted database within NHS Lothian. Personal contact details and CHI numbers will not be entered onto the study database.

The Chief Investigator (DJM) will have access to the data during all stages of the study. The analysis will be performed in secure premises at the University of Edinburgh and NHS Lothian by the Chief Investigator and the assigned statistician. The Chief Investigator will have control of and act as the custodian for the data generated by the study.

For peer review only - http://bmjopen.bmj.com/site/about/guidelines.xhtml

Participants

Consent

Potential study participants will be provided with a written information sheet and a written consent form. In addition, participants will meet with a member of the clinical research facility (CRF) team to receive a full explanation of the nature and purpose of the study, with the opportunity to ask questions. It will be made clear that the individual may withdraw from the study at any time without giving a reason and with no consequence to their current or future care. Recruitment and informed consent will be performed by the investigator/member of the CRF team at the participating site. If individuals wish to participate, they will sign a consent form, countersigned by the investigator/CRF researcher. In addition, consent will be obtained to inform the participants' GP and Consultant in charge of their care.

Patient decision period

Potential participants who have been invited to participate will be given a period of up to 2 days (minimum 1 hour) to read the relevant information sheets, to contact a member of the study team to get further information and ask questions, before they are invited to participate in a conversation and commence the process of fully-informed consent.

Withdrawal of participants

Participants will be withdrawn from the clinical research study in the following circumstances:

i) In cases of withdrawal of informed consent, where capacity exists.
ii) In cases of withdrawal of consent by the participant's representative for adults with incapacity.

Participants who wish to withdraw from the study will be given the option to permit ongoing use of data and samples that have already been collected, and/or future recording and usage of routinely collected clinical data and results. This will be clearly documented on the patient consent form. Patients that become uncontactable will have any previously collected data included in the study unless consent is withdrawn as above.

Benefits to participants

No direct benefit is expected for the participants. We envisage that the results of this study will benefit other patients with AP in the future, and society as a whole by advancing scientific knowledge on this condition. We believe that this study may lead to new strategies in the

For peer review only

treatment of AP which will prevent or ameliorate the impact of organ dysfunction. This will help to maximise the health, and life expectancy, of patients with AP.

Potential burden to participants

The participants will be subjected to peripheral venous blood sampling which may cause discomfort or pain. In order to minimise these effects, blood sampling will be performed by appropriately trained and experienced members of the research team, in adherence to the local guidelines for blood sampling. Where possible, and only if the specific study venepuncture time-points coincide, clinical care samples will be taken at the same time to reduce the number of venepunctures that any one participant sustains.

The collection of urine and stool samples is not expected to cause any harm or discomfort; however, some minimal inconvenience may be caused. In order to address this, a convenient time for the participants and the clinical care team will be chosen, and a full explanation of the purpose of these tests will be provided. The participants' dignity and privacy will be respected at all times.

The six-minute walk-test is not anticipated to cause any harm or discomfort to participants. Pulse wave analysis is not expected to cause any significant adverse effects. Inhaled salbutamol may cause a very slight peripheral tremor, but no other expected harm or discomfort.

CPEX testing is associated with a very low risk of serious complications; however, this is related to the severity of co-morbid disease such as coronary artery disease (CAD)[25]. For those participants with identified CAD, the opinion of a cardiologist will be sought on the suitability of the participant for this test.

Assessment of pulmonary function using spirometry is not known to be associated with any significant adverse events[31]. Participants may experience some discomfort or dizziness during the test due to the requirement for complete exhalation[32]. Spirometry is associated with increased myocardial strain, and a rise in intrathoracic, intraabdominal and intraocular pressures, and consequently will not be performed on participants with the following: recent head/neck, thoracic or abdominal surgery, recent myocardial infarction, retinal detachment, resting pulse greater than 120bpm, pregnant, severe heart failure or arrhythmia, pneumothorax, aortic or cerebral aneurysm[33].

For peer review only - http://bmjopen.bmj.com/site/about/guidelines.xhtml

MRI scans are not expected to pose risks to the majority of participants, in the absence of known contraindications. Participants will be assessed prior to the scan for any potential contraindications to the procedure such as mechanical heart valves, pacemakers, metallic prostheses and cochlear implants[34].

ETHICS AND DISSEMINATION

This study has been reviewed as IRAS Number 178615 by the South-East Scotland Research Ethics Committee under the application numbers (REC Number) 16/SS/0065 and has been given a favourable opinion. NHS Scotland R&D approval have been granted. The study sponsor is NHS Lothian/University of Edinburgh ACCORD. The trial is registered at ClinicalTrials.gov (NCT03342716).

Results of the study will be presented at local, national and international medical meetings. The findings of the study will be published in peer-reviewed medical/scientific journals and made open-access on acceptance. If appropriate, the results of the study will be disseminated via press releases by the University of Edinburgh. Information may also be disseminated to the general public via public engagement and community outreach programmes.

Patient and Public Involvement statement: Patients who have had an episode of AP and who participate in the APPLe (acute pancreatitis patient liaison group) were invited to input and contributed to the concepts and the trial design.

VERSIONING: This paper is based on the full protocol V11, dated 23rd September 2019

AUTHOR CONTRIBUTIONS

DM had the idea and wrote the funding application with statistics input from CG. DJM, CG and JB developed and drafted the clinical study protocol and ethics application. DM, JB, MG, KW, EW, LM, EC, ET, AS, DD, RM, SC and other RESORP team initiated the project. AS, RM, DM drafted the manuscript. All authors contributed to and approved the final version of the manuscript.

FUNDING

For peer review only

435 This study is funded by the United Kingdom Medical Research Council through a Senior Clinical Fellowship to Damian J. Mole. Ref: MR/P008887/1.

438 **DECLARATION OF INTEREST**

439 No individual has an actual or perceived conflict of interest with this study. DJM wishes to declare that he holds a senior position in Kynos Therapeutics Ltd, a private company developing medicines for systemic inflammation and cancer, and that he has previously received funding for collaborative studies on AP from GSK.

For peer review only

REFERENCES

1. Yadav D, Lowenfels AB. The epidemiology of pancreatitis and pancreatic cancer. *Gastroenterology* 2013;144(6):1252-61. doi: 10.1053/j.gastro.2013.01.068 [published Online First: 2013/04/30]

2. Banks PA. Epidemiology, natural history, and predictors of disease outcome in acute and chronic pancreatitis. *Gastrointest Endosc* 2002;56(6 Suppl):S226-30. doi: 10.1067/mge.2002.129022 [published Online First: 2002/11/26]

3. Lankisch PG, Apte M, Banks PA. Acute pancreatitis. *Lancet* 2015;386(9988):85-96. doi: 10.1016/S0140-6736(14)60649-8 [published Online First: 2015/01/27]

4. McKay CJ, Evans S, Sinclair M, et al. High early mortality rate from acute pancreatitis in Scotland, 1984-1995. *Br J Surg* 1999;86(10):1302-5. doi: 10.1046/j.1365-2168.1999.01246.x [published Online First: 1999/10/30]

5. Roberts SE, Williams JG, Meddings D, et al. Incidence and case fatality for acute pancreatitis in England: geographical variation, social deprivation, alcohol consumption and aetiology--a record linkage study. *Aliment Pharmacol Ther* 2008;28(7):931-41. doi: 10.1111/j.1365-2036.2008.03809.x [published Online First: 2008/07/24]

6. Skouras C, Hayes AJ, Williams L, et al. Early organ dysfunction affects long-term survival in acute pancreatitis patients. *HPB (Oxford)* 2014;16(9):789-96. doi: 10.1111/hpb.12259 [published Online First: 2014/04/10]

7. Mole DJ, Gungabissoon U, Johnston P, et al. Identifying risk factors for progression to critical care admission and death among individuals with acute pancreatitis: a record linkage analysis of Scottish healthcare databases. *BMJ open* 2016;6(6):e011474. doi: 10.1136/bmjopen-2016-011474 [published Online First: 2016/06/18]

8. Dellinger EP, Forsmark CE, Layer P, et al. Determinant-based classification of acute pancreatitis severity: an international multidisciplinary consultation. *Ann Surg* 2012;256(6):875-80. doi: 10.1097/SLA.0b013e318256f778 [published Online First: 2012/06/28]

9. Banks PA, Bollen TL, Dervenis C, et al. Classification of acute pancreatitis--2012: revision of the Atlanta classification and definitions by international consensus. *Gut*

For peer review only - http://bmjopen.bmj.com/site/about/guidelines.xhtml

2013;62(1):102-11. doi: 10.1136/gutjnl-2012-302779 [published Online First: 2012/10/27]

10. Johnson CD, Abu-Hilal M. Persistent organ failure during the first week as a marker of fatal outcome in acute pancreatitis. *Gut* 2004;53(9):1340-4. doi: 10.1136/gut.2004.039883 [published Online First: 2004/08/13]

11. Garg PK, Singh VP. Organ Failure Due to Systemic Injury in Acute Pancreatitis. *Gastroenterology* 2019;156(7):2008-23. doi: 10.1053/j.gastro.2018.12.041 [published Online First: 2019/02/16]

12. al. Ne. Molecular patterns in acute pancreatitis reflect generalisable endotypes of the host response to systemic injury in humans., 2020.

13. Skouras C, Hayes A, Williams I. Early organ dysfunction affects long term survival in acute pancreatitis patients. *HPB* 2013;16(9):789-96.

14. Nøjgaard C. Prognosis of acute and chronic pancreatitis: a 30 year follow up of a Danish cohort. *Dan Med Bull* 2010;57(12):B4228.

15. Lankisch P, Breuer N, Bruns A. Natural history of acute pancreatitis: a long-term population-based study. *Am J Gastroenterol* 2009;104(11):2797-805.

16. Cavestro G, Leandro G, Di Leo M. A single-centre propsective, cohort study of the natural history of acute pancreatitis. *Dig Liver Dis* 2015;47(3):205-10.

17. Appelros S, Lingren S, Borgstrom A. Short and long-term outcome of severe acute pancreatitis. *Eur J Surg* 2001;167(4):281-86.

18. Halonen K, Pettila V, Leppaniemi A. Long-term health-related quality of life in survivors of severe acute pancreatitis. *Intensive Care Med* 2003;29(5):782-86.

19. Ciesla N, Dinglas V, Fan E, et al. Manual Muscle Testing: A Method of Measuring Extremity Muscle Strength Applied to Critically Ill Patients. *JoVE* 2011(50):e2632. doi: doi:10.3791/2632

20. Sway balance 2020 [cited 2020 20 February]. Available from: http://swaymedical.com/system/balance.

21. Amick RZ, Chaparro A, Patterson JA. Test-Retest Reliability of the sway Balance Mobile Application. *Journal of Mobile Technology in Medicine* 2015;4(2):40-47. doi: 10.7309/jmtm.4.2.6

22. Six-minute walk test 2020 [cited 2020 20 February]. Available from: http://www.pulmonaryrehab.com.au/index.asp?page=19.

For peer review only

For peer review only - http://bmjopen.bmj.com/site/about/guidelines.xhtml

23. Guidelines for the measurement of respiratory function. Recommendations of the British Thoracic Society and the Association of Respiratory Technicians and Physiologists. *Respir Med* 1994;88(3):165-94. [published Online First: 1994/03/01]

24. K W, JE H, DY S, et al. Principles of Exercise Testing and Interpretation. Philadelphia, PA: Lippincott Williams & Wilkins 2005:133-156.

25. American Thoracic S, American College of Chest P. ATS/ACCP Statement on cardiopulmonary exercise testing. *Am J Respir Crit Care Med* 2003;167(2):211-77. doi: 10.1164/rccm.167.2.211 [published Online First: 2003/01/14]

26. Ewald N, Kaufmann C, Raspe A, et al. Prevalence of diabetes mellitus secondary to pancreatic diseases (type 3c). *Diabetes Metab Res Rev* 2012;28(4):338-42. doi: 10.1002/dmrr.2260 [published Online First: 2011/11/29]

27. Woodmansey C, McGovern AP, McCullough KA, et al. Incidence, Demographics, and Clinical Characteristics of Diabetes of the Exocrine Pancreas (Type 3c): A Retrospective Cohort Study. *Diabetes Care* 2017;40(11):1486-93. doi: 10.2337/dc17-0542 [published Online First: 2017/09/02]

28. Banerjee R, Pavlides M, Tunnicliffe EM, et al. Multiparametric magnetic resonance for the non-invasive diagnosis of liver disease. *J Hepatol* 2014;60(1):69-77. doi: 10.1016/j.jhep.2013.09.002 [published Online First: 2013/09/17]

29. Harris PA, Taylor R, Minor BL, et al. The REDCap consortium: Building an international community of software platform partners. *J Biomed Inform* 2019;95:103208. doi: 10.1016/j.jbi.2019.103208 [published Online First: 2019/05/13]

30. Harris PA, Taylor R, Thielke R, et al. Research electronic data capture (REDCap)--a metadata-driven methodology and workflow process for providing translational research informatics support. *J Biomed Inform* 2009;42(2):377-81. doi: 10.1016/j.jbi.2008.08.010 [published Online First: 2008/10/22]

31. Lin K, Watkins B, Johnson T, et al. Screening for chronic obstructive pulmonary disease using spirometry: summary of the evidence for the U.S. Preventive Services Task Force. *Ann Intern Med* 2008;148(7):535-43. doi: 10.7326/0003-4819-148-7-200804010-00213 [published Online First: 2008/03/05]

32. Buist AS, Vollmer WM, Sullivan SD, et al. The Burden of Obstructive Lung Disease Initiative (BOLD): rationale and design. *COPD* 2005;2(2):277-83. [published Online First: 2006/12/02]

For peer review only - http://bmjopen.bmj.com/site/about/guidelines.xhtml

33. Coates AL, Graham BL, McFadden RG, et al. Spirometry in primary care. *Can Respir J* 2013;20(1):13-21. doi: 10.1155/2013/615281 [published Online First: 2013/03/05]

34. Dill T. Contraindications to magnetic resonance imaging: non-invasive imaging. *Heart* 2008;94(7):943-8. doi: 10.1136/hrt.2007.125039 [published Online First: 2008/06/17]

For peer review only

SPIRIT

STANDARD PROTOCOL ITEMS: RECOMMENDATIONS FOR INTERVENTIONAL TRIALS

Study Protocol for RESORP – Resolution of Organ Injury in Acute Pancreatitis – an observational prospective cohort study

SPIRIT 2013 Checklist: Recommended items to address in a clinical trial protocol and related documents*

Section/item	Item No	Description
**Administrative information**		
Title	1	Descriptive title identifying the study design, population, interventions, and, if applicable, trial acronym - yes
Trial registration	2a	Trial identifier and registry name. If not yet registered, name of intended registry - yes
	2b	All items from the World Health Organization Trial Registration Data Set
Protocol version	3	Date and version identifier – yes, V11 23/9/19
Funding	4	Sources and types of financial, material, and other support – declared, with ref numbers
Roles and responsibilities	5a	Names, affiliations, and roles of protocol contributors - yes
	5b	Name and contact information for the trial sponsor - yes
	5c	Role of study sponsor and funders, if any, in study design; collection, management, analysis, and interpretation of data; writing of the report; and the decision to submit the report for publication, including whether they will have ultimate authority over any of these activities - yes
	5d	Composition, roles, and responsibilities of the coordinating centre, steering committee, endpoint adjudication committee, data management team, and other individuals or groups overseeing the trial, if applicable (see Item 21a for data monitoring committee) - yes
**Introduction**		
Background and rationale	6a	Description of research question and justification for undertaking the trial, including summary of relevant studies (published and unpublished) examining benefits and harms for each intervention - yes
	6b	Explanation for choice of comparators – not applicable
Objectives	7	Specific objectives or hypotheses – clearly stated

For peer review only

Trial design	8	Description of trial design including type of trial (eg, parallel group, crossover, factorial, single group), allocation ratio, and framework (eg, superiority, equivalence, noninferiority, exploratory) - done

Methods: Participants, interventions, and outcomes

Study setting	9	Description of study settings (eg, community clinic, academic hospital) and list of countries where data will be collected. Reference to where list of study sites can be obtained – clearly stated
Eligibility criteria	10	Inclusion and exclusion criteria for participants. If applicable, eligibility criteria for study centres and individuals who will perform the interventions (eg, surgeons, psychotherapists) – clearly stated
Interventions	11a	Interventions for each group with sufficient detail to allow replication, including how and when they will be administered – not applicable
	11b	Criteria for discontinuing or modifying allocated interventions for a given trial participant (eg, drug dose change in response to harms, participant request, or improving/worsening disease) – not applicable
	11c	Strategies to improve adherence to intervention protocols, and any procedures for monitoring adherence (eg, drug tablet return, laboratory tests) – not applicable
	11d	Relevant concomitant care and interventions that are permitted or prohibited during the trial– not applicable
Outcomes	12	Primary, secondary, and other outcomes, including the specific measurement variable (eg, systolic blood pressure), analysis metric (eg, change from baseline, final value, time to event), method of aggregation (eg, median, proportion), and time point for each outcome. Explanation of the clinical relevance of chosen efficacy and harm outcomes is strongly recommended– clearly stated
Participant timeline	13	Time schedule of enrolment, interventions (including any run-ins and washouts), assessments, and visits for participants. A schematic diagram is highly recommended (see Figure) – clearly stated
Sample size	14	Estimated number of participants needed to achieve study objectives and how it was determined, including clinical and statistical assumptions supporting any sample size calculations– clearly stated
Recruitment	15	Strategies for achieving adequate participant enrolment to reach target sample size– clearly stated

Methods: Assignment of interventions (for controlled trials)

Allocation:

For peer review only - http://bmjopen.bmj.com/site/about/guidelines.xhtml

Sequence generation	16a	Method of generating the allocation sequence (eg, computer-generated random numbers), and list of any factors for stratification. To reduce predictability of a random sequence, details of any planned restriction (eg, blocking) should be provided in a separate document that is unavailable to those who enrol participants or assign interventions – not applicable
Allocation concealment mechanism	16b	Mechanism of implementing the allocation sequence (eg, central telephone; sequentially numbered, opaque, sealed envelopes), describing any steps to conceal the sequence until interventions are assigned – not applicable
Implementation	16c	Who will generate the allocation sequence, who will enrol participants, and who will assign participants to interventions – not applicable
Blinding (masking)	17a	Who will be blinded after assignment to interventions (eg, trial participants, care providers, outcome assessors, data analysts), and how – not applicable
	17b	If blinded, circumstances under which unblinding is permissible, and procedure for revealing a participant's allocated intervention during the trial – not applicable
**Methods: Data collection, management, and analysis**		
Data collection methods	18a	Plans for assessment and collection of outcome, baseline, and other trial data, including any related processes to promote data quality (eg, duplicate measurements, training of assessors) and a description of study instruments (eg, questionnaires, laboratory tests) along with their reliability and validity, if known. Reference to where data collection forms can be found, if not in the protocol – clearly stated
	18b	Plans to promote participant retention and complete follow-up, including list of any outcome data to be collected for participants who discontinue or deviate from intervention protocols – clearly stated
Data management	19	Plans for data entry, coding, security, and storage, including any related processes to promote data quality (eg, double data entry; range checks for data values). Reference to where details of data management procedures can be found, if not in the protocol – clearly stated
Statistical methods	20a	Statistical methods for analysing primary and secondary outcomes. Reference to where other details of the statistical analysis plan can be found, if not in the protocol – clearly stated
	20b	Methods for any additional analyses (eg, subgroup and adjusted analyses) – clearly stated
	20c	Definition of analysis population relating to protocol non-adherence (eg, as randomised analysis), and any statistical methods to handle missing data (eg, multiple imputation) – clearly stated

For peer review only - http://bmjopen.bmj.com/site/about/guidelines.xhtml

Methods: Monitoring

Data monitoring	21a	Composition of data monitoring committee (DMC); summary of its role and reporting structure; statement of whether it is independent from the sponsor and competing interests; and reference to where further details about its charter can be found, if not in the protocol. Alternatively, an explanation of why a DMC is not needed – not applicable
	21b	Description of any interim analyses and stopping guidelines, including who will have access to these interim results and make the final decision to terminate the trial – not applicable
Harms	22	Plans for collecting, assessing, reporting, and managing solicited and spontaneously reported adverse events and other unintended effects of trial interventions or trial conduct – clearly stated
Auditing	23	Frequency and procedures for auditing trial conduct, if any, and whether the process will be independent from investigators and the sponsor– clearly stated

Ethics and dissemination

Research ethics approval	24	Plans for seeking research ethics committee/institutional review board (REC/IRB) approval– obtained
Protocol amendments	25	Plans for communicating important protocol modifications (eg, changes to eligibility criteria, outcomes, analyses) to relevant parties (eg, investigators, REC/IRBs, trial participants, trial registries, journals, regulators) – clearly stated
Consent or assent	26a	Who will obtain informed consent or assent from potential trial participants or authorised surrogates, and how (see Item 32) – clearly stated
	26b	Additional consent provisions for collection and use of participant data and biological specimens in ancillary studies, if applicable– clearly stated
Confidentiality	27	How personal information about potential and enrolled participants will be collected, shared, and maintained in order to protect confidentiality before, during, and after the trial– clearly stated
Declaration of interests	28	Financial and other competing interests for principal investigators for the overall trial and each study site– clearly stated
Access to data	29	Statement of who will have access to the final trial dataset, and disclosure of contractual agreements that limit such access for investigators– clearly stated

For peer review only - http://bmjopen.bmj.com/site/about/guidelines.xhtml

Ancillary and post-trial care	30	Provisions, if any, for ancillary and post-trial care, and for compensation to those who suffer harm from trial participation – not applicable
Dissemination policy	31a	Plans for investigators and sponsor to communicate trial results to participants, healthcare professionals, the public, and other relevant groups (eg, via publication, reporting in results databases, or other data sharing arrangements), including any publication restrictions – clearly stated
	31b	Authorship eligibility guidelines and any intended use of professional writers– clearly stated
	31c	Plans, if any, for granting public access to the full protocol, participant-level dataset, and statistical code– clearly stated
**Appendices**		
Informed consent materials	32	Model consent form and other related documentation given to participants and authorised surrogates – not included
Biological specimens	33	Plans for collection, laboratory evaluation, and storage of biological specimens for genetic or molecular analysis in the current trial and for future use in ancillary studies, if applicable– clearly stated

*It is strongly recommended that this checklist be read in conjunction with the SPIRIT 2013 Explanation & Elaboration for important clarification on the items. Amendments to the protocol should be tracked and dated. The SPIRIT checklist is copyrighted by the SPIRIT Group under the Creative Commons "Attribution-NonCommercial-NoDerivs 3.0 Unported" license.

For peer review only

Study Protocol for RESORP – Resolution of Organ Injury in Acute Pancreatitis – an observational prospective cohort study.

Journal:	*BMJ Open*
Manuscript ID	bmjopen-2020-040200.R1
Article Type:	Protocol
Date Submitted by the Author:	05-Aug-2020
Complete List of Authors:	Sherif, Ahmed; The University of Edinburgh, Clinical Surgery<br>McFadyen, Rory; The University of Edinburgh,<br>Boyd, Julia; Edinburgh Clinical Trials Unit<br>Ventre, Chiara; The University of Edinburgh,<br>Glenwright, Margaret; NHS Lothian<br>Walker, Kim; NHS Lothian<br>Zheng, Xiaozhong; University of Edinburgh, Centre for Inflammation Research<br>White, Audrey; NHS Lothian<br>McFadyen, Laura; NHS Lothian<br>Connon, Emma; NHS Lothian<br>Damaskos, Dimitrios<br>Steven, Michelle; University of Edinburgh, Edinburgh Clinical Trials Unit<br>Wackett, Anthony<br>Thomson, Euan; NHS Lothian<br>Cameron, David; NHS Lothian<br>Semple, Scott<br>Morris, David<br>Clark-Stewart, Saskia; NHS Lothian<br>Graham, Catriona<br>Mole, Damian; University of Edinburgh MRC Centre for Inflammation Research, ; The University of Edinburgh, Clinical Surgery
**Primary Subject Heading**:	Surgery
Secondary Subject Heading:	Gastroenterology and hepatology, Intensive care, Nutrition and metabolism, Diabetes and endocrinology, Emergency medicine
Keywords:	Pancreatic disease < GASTROENTEROLOGY, Adult intensive & critical care < ANAESTHETICS, General diabetes < DIABETES & ENDOCRINOLOGY

SCHOLARONE™ Manuscripts

I, the Submitting Author has the right to grant and does grant on behalf of all authors of the Work (as defined in the below author licence), an exclusive licence and/or a non-exclusive licence for contributions from authors who are: i) UK Crown employees; ii) where BMJ has agreed a CC-BY licence shall apply, and/or iii) in accordance with the terms applicable for US Federal Government officers or employees acting as part of their official duties; on a worldwide, perpetual, irrevocable, royalty-free basis to BMJ Publishing Group Ltd ("BMJ") its licensees and where the relevant Journal is co-owned by BMJ to the co-owners of the Journal, to publish the Work in this journal and any other BMJ products and to exploit all rights, as set out in our licence.

The Submitting Author accepts and understands that any supply made under these terms is made by BMJ to the Submitting Author unless you are acting as an employee on behalf of your employer or a postgraduate student of an affiliated institution which is paying any applicable article publishing charge ("APC") for Open Access articles. Where the Submitting Author wishes to make the Work available on an Open Access basis (and intends to pay the relevant APC), the terms of reuse of such Open Access shall be governed by a Creative Commons licence – details of these licences and which Creative Commons licence will apply to this Work are set out in our licence referred to above.

Other than as permitted in any relevant BMJ Author's Self Archiving Policies, I confirm this Work has not been accepted for publication elsewhere, is not being considered for publication elsewhere and does not duplicate material already published. I confirm all authors consent to publication of this Work and authorise the granting of this licence.

For peer review only

Study Protocol for RESORP – Resolution of Organ Injury in Acute Pancreatitis – an observational prospective cohort study

Authors

Ahmed E Sherif[1*], Rory McFadyen[1*], Julia Boyd[2*], Chiara Ventre[1], Margaret Glenwright[3], Kim Walker[3], Xiaozhong Zheng[4], Audrey White[3], Laura McFadyen[3], Emma Connon[3], Dimitrios Damaskos[1], Michelle Steven[2], Anthony Wackett[2], Euan Thomson[5], David C Cameron[5], Scott Semple[6], David Morris[4], Saskia Clark-Stewart[1], Catriona Graham[7], and Damian J Mole[1,4**] on behalf of the RESORP research team

*indicates joint 1st authors

** indicates corresponding author

Affiliations

[1] Clinical Surgery, University of Edinburgh, Royal Infirmary of Edinburgh, 51 Little France Crescent, Edinburgh, EH16 4TJ, UK

[2] Edinburgh Clinical Trials Unit, Nine Bioquarter, Edinburgh EH16 4UX, UK

[3] Clinical Research Facility, NHS Lothian, Edinburgh, EH16 4TJ, UK

[4]Centre for Inflammation Research, University of Edinburgh, Queen's Medical Research Institute, 47 Little France Crescent, Edinburgh, EH16 4TJ, UK

[5] Department of Anaesthesia, NHS Lothian, Edinburgh, EH16 4TJ, UK

[6]Centre for Cardiovascular Science, University of Edinburgh, Queen's Medical Research Institute, Edinburgh BioQuarter, 47 Little France Crescent, Edinburgh, EH16 4TJ, UK

[7]Epidemiology and Statistics Core, Edinburgh Clinical Research Facility, University of Edinburgh, Western General Hospital, Crewe Road South, Edinburgh EH4 2XU

Correspondence to: Prof Damian J Mole; damian.mole@ed.ac.uk, +44 (131) 242 9100

For peer review only

ABSTRACT

Introduction: Survivors of acute pancreatitis (AP) have shorter overall survival and increased incidence of new-onset cardiovascular, respiratory, liver and renal disease, diabetes mellitus and cancer compared to the general population, but the mechanisms that explain this are yet to be elucidated. Our aim is to characterise the precise nature and extent of organ dysfunction following an episode of AP.

Methods and Analysis

This is an observational prospective cohort study in a single centre comprising a University hospital with an acute and emergency receiving unit and clinical research facility. Participants will be adult patient admitted with AP. Participants will undergo assessment at recruitment, 3 months and 3 years. At each time point, multiple biochemical and/or physiological assessments to measure cardiovascular, respiratory, liver, renal and cognitive function, diabetes mellitus, and quality of life. Recruitment was from 30th November 2017 to 31st May 2020; last follow-up measurements is due 31st May 2023. The primary outcome measure is the incidence of new-onset type 3c diabetes mellitus during follow-up. Secondary outcome measures include: quality of life analyses (SF-36, GIQLI); Montreal cognitive assessment; organ system physiological performance; multiomics predictors of AP severity, detection of premature cellular senescence. In a nested cohort within the main cohort, individuals may also consent to multiparameter MRI scan, echocardiography, pulmonary function testing, cardiopulmonary exercise testing and pulse-wave analysis.

Ethics and dissemination: This study has received the following approvals: UK IRAS Number 178615; South-east Scotland Research Ethics Committee number 16/SS/0065. Results will be made available to AP survivors, caregivers, funders and other researchers. Publications will be open-access.

Trial registration: ClinicalTrials.gov Identifier (NCT03342716) and ISRCTN 50581876; Pre-results

Strengths and limitations

Strengths

- Prospective cohort study with molecular mechanistic observations.
- In-depth clinical and physiological annotation of multiomics data.

For peer review only - http://bmjopen.bmj.com/site/about/guidelines.xhtml

Limitations

- Drop-out rates in long term prospective AP studies is largely unknown.
- Potential participants with severe AP may lack capacity for consent and recruitment may therefore be slower in this group.

Keywords: long-term outcome; acute pancreatitis; organ failure; post-inflammatory premature senescence

Word Count: 3709

For peer review only

INTRODUCTION

Acute pancreatitis (AP) is an inflammatory disease of the pancreas usually triggered by gallstones or excess alcohol consumption. The incidence of AP ranges from 13 to 45 per 100,00 individuals, is increasing[1], and varies between nations. For example, USA, Finland, Scotland and Germany report higher incidences than England and The Netherlands[2-5]. AP triggers a cascade of inflammatory events causing cell damage and release of damage-associated molecular patterns that trigger inflammation and cytokine release, initiating a systemic inflammatory response resulting in multiple organ dysfunction syndrome (MODS) in 1 in 5 individuals. AP-MODS has a 21% fatality rate during the index episode, and overall in-hospital mortality is 5.2% in Scotland[6 7]. AP is graded into mild, moderate and severe, based on local and systemic determinants of severity as per the most recent AP classifications (Determinant-based Classification[8] and Revised Atlanta Classification[9]). Organ failure in AP most commonly affects the respiratory, renal and cardiovascular systems, but can involve any system[10]. The pattern of organ failure is difficult to predict and a high level of inter-individual variance is seen, regardless of the aetiology. The molecular mechanisms that explain this inter-individual variation in local and systemic inflammatory processes in AP remain unidentified [11]. Multiomics analysis of serial time course blood samples has defined molecular patterns in human AP, called endotypes, but the precise relevance of these endotypes to clinical and therapeutic decision-making is not yet determined[12].

Recently published data from a retrospective analysis of patients with AP has shown that the early development of MODS is associated with an increased mortality rate up to 10 years after the index presentation[13]. This strongly suggests that MODS in acute pancreatitis (AP-MODS) has a persistent and deleterious impact on patients' physiological status, though the exact nature of this pathology remains to be characterised. Existing studies indicate that most deaths are the result of cardiovascular, pulmonary, digestive or malignant disease[14-17]. In addition, the new onset of diabetes (Type 3c DM) or impaired glucose tolerance, peripheral neuropathy and exocrine dysfunction have been documented as sources of morbidity in the years following the index attack of SAP[17 18]. Patients with alcohol-induced pancreatitis may be at particularly increased risk of progression to chronic pancreatitis[15], however the development of long term complications is likely to be influenced by multiple, and as-yet unconfirmed, factors.

For peer review only - http://bmjopen.bmj.com/site/about/guidelines.xhtml

The aim of this observational clinical cohort study is to characterise the nature and extent of the pathophysiological impact of severe AP on organ function over the first 3 years after the index AP episode and define the long-term deleterious effect of severe AP. This will be achieved by prospectively measuring organ system function in patients recruited during a hospital admission with AP and at set time intervals thereafter. We will obtain an in-depth assessment of patients' health at presentation, and at 3 months and 36 months after the first episode of AP using markers of organ function and/or disease in the peripheral blood. In a nested cohort within the main study cohort, we will conduct cardiorespiratory evaluation tests (including exercise testing), specialised blood tests of the immune system, tests for precision medicine, and imaging to assess structure and function of key organ systems.

The results of this study will inform the design of future interventions designed to improve the long-term prognosis of patients.

METHODS

Ethical approval

The study will be conducted in accordance with the principles of the International Conference on Harmonisation Tripartite Guideline for Good Clinical Practice (ICH GCP). This study has been reviewed and been given a favourable opinion by the South-East Scotland Research Ethics Committee, under the application numbers: REC Number 16/SS/0065; IRAS Number 178615; and R&D approval Lothian R&D Number 2016/0152.

Trials registration

This study was prospectively registered with www.clinicaltrials.gov NCT03342716 and with ISRCTN 50581876.

Study design

This is an observational prospective nested cohort study. The overall cohort is the population presenting with AP that meets the eligibility criteria. For the nested cohort of the study, a stratified block design is to be used to recruit equivalent numbers of patients with AP-MODS versus AP without MODS.

For peer review only

Setting

Single centre academic hospital setting – Royal Infirmary of Edinburgh, NHS Lothian and the Edinburgh Clinical Research Facility, Royal Infirmary of Edinburgh, UK.

Duration

Recruitment started on 30th November 2017 and is scheduled to end on 31st May 2020. The cohort assessment will comprise of 3 study visits. In-depth assessments of a participant's health at presentation, at 3 months and at 36 months follow-up study visit will be obtained. Additional cardiorespiratory evaluation tests, specialised blood tests of the immune system, tests for precision medicine, and imaging to assess structure and function of key organ systems will be conducted in a nested cohort of participants.

Intervention

This is a prospective study examining patient outcomes and trends in patients' physiological status after an index event. Therefore, no intervention or randomisation is proposed.

Measurements

Primary endpoint

- Overall cohort: to define the incidence of new-onset type 3c diabetes mellitus in patients with AP, compared to the age-matched population of Scotland.
- Nested cohort: to define the baseline change in pancreatic fibrosis index measured by multi-parameter MRI between 3 and 36 months after recruitment.

Secondary endpoints

Secondary end-points in the study will be experimental and observational, and will define explicitly, or be preliminary exploratory studies investigating:

i) Genomic predictors of AP severity and resolution of inflammation.
ii) miRNA signatures of disease severity and resolution.
iii) Metabolomic profiling of AP resolution, and development of local and systemic complications.
iv) Premature cellular senescence as a pathological consequence of AP-MODS.

For peer review only - http://bmjopen.bmj.com/site/about/guidelines.xhtml

v) Alteration in immune cell subsets and phenotype as a long-term response to AP-MODS.

vi) Cardiovascular and respiratory dysfunction as a legacy of endothelial injury during AP-MODS.

Summary of clinical data to be collected

All participants in the overall and nested cohorts will have the following data collected:

i) Demographic and Clinical Background Information.

ii) Information regarding the acute presentation.

iii) Organ function assessment at recruitment, 3- and 36-months follow-up study visit:

a. Overall cohort:

   i. Full peripheral venous blood profiling, including cardiac biomarkers, standard biochemistry profiling, and samples retained for miRNA profiling, cytokines, telomere length, metabolomic profiling, proteomic profiling, transcriptomic profiling, genomic profiling, leukocyte subset analysis by flow cytometry.

   ii. Biochemical markers of organ function in urine and samples retained.

   iii. Pancreatic exocrine function test in stool (faecal elastase).

   iv. Nutritional assessment.

   v. Oral glucose tolerance test at 3- and 36-month follow-up study visit (with the exception of insulin dependent diabetics who will have random blood glucose measured only).

   vi. 12 lead electrocardiogram (ECG), blood pressure.

   vii. Peripheral SpO2.

   viii. Sway balance app non-invasive muscle function tests, 6-minute walk test.

   ix. Self-administered Patient Questionnaire

      1. Gastrointestinal Quality of Life Index (GIQLI).
      2. SF-12 Quality of Life.
      3. Montreal Cognitive Assessment.

b. Nested cohorts:

For peer review only

i. Pulse wave analysis (with salbutamol inhaler); transthoracic echocardiography.
ii. Spirometry and gas transfer.
iii. Cardiopulmonary exercise (CPEX) testing.
iv. Multiparameter magnetic resonance imaging (MRI) assessment of liver and pancreas.

Venous blood, urine and stool sampling

Blood samples will be obtained from each study participant at each of the three study visits for organ function and specialist assays. A urine sample and a stool sample will be obtained at each of the 3 visits.

Nutritional assessment

Participants will have their height and weight measured in order to calculate their Body Mass Index (BMI), as well as bioimpedance analysis +/- upper arm anthropometry to assess percentage body fat and lean tissue mass. Participants will also be asked to keep a 24-hour food diary to assess whether their calorie and protein intake is sufficient for their nutritional needs at all 3 study visits. Trace element, including zinc, selenium and B12 levels and other relevant micronutrients will be measured in peripheral venous blood.

Oral glucose tolerance test

At the 3- and 36-month follow-up study visits an oral glucose tolerance test will be performed. Participants attending the research site for physiological testing will be asked to attend in a fasted state. On arrival participants will have the baseline blood sample taken (using the same venepuncture as other tests above as appropriate), be given a standard 75g anhydrous glucose in cold water or Polycal ® (113mL) which is more palatable and will be followed by 150mL water (total volume should be 250-300mL). After 120 minutes, a second blood sample will be taken (using the same venepuncture as other tests above as appropriate) to measure blood glucose.

An oral glucose tolerance test will not be performed in participants with insulin dependent diabetes. Instead a random glucose sample will be taken at these visits.

Non-invasive muscle function testing, Sway balance test, 6-minute walk test

For peer review only

For peer review only - http://bmjopen.bmj.com/site/about/guidelines.xhtml

Manual Muscle Testing will be performed once per visit to assess muscle strength. This will follow structured protocols used to assess muscle strength in survivors of critical illness[19]. The strength of muscles involved in shoulder abduction, elbow flexion, wrist extension, hip flexion, knee extension and ankle dorsiflexion will be graded according to the Medical Research Council (MRC) muscle strength scoring system.

Participants will undergo an assessment of balance and postural stability at each visit. Testing will follow a validated protocol using the SWAY Balance mobile testing system[20 21]. Participants will be asked to hold a mobile device against their chest whilst completing a series of stances: standing with both feet together, standing with one foot behind the other, and standing on each leg in turn[21]. Each stance is held for 10 seconds with the eyes closed. The SWAY Balance application will generate a balance score, based on detection of alterations in position during each stance.

A six-minute walk test will be done, according to standard protocols[22].

Multiparameter magnetic resonance imaging (Liver & Pancreas)

A multiparameter MRI scan will be performed on each visit for the nested cohort to measure lipid, iron load and fibrosis in liver and pancreas according to protocols in development at CRIC (Clinical Research Imaging Centre). The total duration of scan will be approximately 30 minutes.

Cardiovascular Analysis

Nested cohort participants will undergo pulse wave analysis (PWA) at the radial artery using a non-invasive tonometer to measure wave reflection, pulse pressure and derived arterial stiffness with the augmentation index at 75 bpm and the administration of two puffs of inhaled salbutamol (200 micrograms each puff) via a spacer device. Transthoracic echocardiography will be performed to measure longitudinal shortening of the myocardium.

Pulmonary Function Testing and Gas Transfer

Spirometry will be undertaken in accordance with guidance from the British Thoracic Society for the nested cohort group[23]. All measurement will be made with participants seated. FEV1 and FVC will be recorded as the best of 3 consistent readings, and compared to the predicted normal values for the participant's height, age, gender and ethnicity. Flow volume measurements will also be obtained. Lung volumes will be measured using the steady state

For peer review only - http://bmjopen.bmj.com/site/about/guidelines.xhtml

method[23]. Gas Transfer/Diffusion Capacity will be measured using the single breath carbon monoxide transfer factor method[23].

Cardiopulmonary Exercise Testing (CPEX)

CPEX testing will be carried out on day 2 of patient assessment at all visits for nested cohort participants. Participants will be asked not to smoke for 8 hours prior to the test, and to abstain from other strenuous exercise on the same day.

Supervised CPEX testing will be conducted following a standardised incremental protocol on a magnetically braked cycle ergometer. Work-load ramp will be determined using a standard formula[24]. Testing will be continued until symptomatic limitation, volitional termination, failure to maintain required cadence or if other early termination conditions are met[25]. Breath by breath O_2 and CO_2 analysis will be performed using a fully calibrated metabolic cart. 12-lead ECG and O_2 saturations will be monitored continuously in the pre-test, test and recovery phase. Non-invasive blood pressure will be recorded at rest and at 3-minute intervals during test and recovery phase. The anaerobic threshold (AT) will be independently determined by two assessors using the V-slope method.

Recruitment

All adult patients that meet the inclusion criteria at the participating sites will be recruited where possible. Patients admitted with an elevated serum amylase will be identified and members of the clinical research team will screen these patients for a diagnosis of AP using the *TRAKCare®* patient management system (InterSystems, Massachusetts, USA). Potential participants will be approached by a member of the direct care team to confirm if they are willing to discuss the research with a member of the research team. If the individual agrees, they will be contacted by a member of the research team during their inpatient stay. If potential participants are discharged before they can be approached, they will not be included in the study

Inclusion criteria

The following inclusion criteria will apply to all patients:

i) Confirmed clinical or radiological diagnosis of acute pancreatitis with an elevated serum amylase greater than 300 U/L.

For peer review only - http://bmjopen.bmj.com/site/about/guidelines.xhtml

ii) For the potential clinical diagnosis of acute pancreatitis an appropriate clinical history based on compatible clinical features, will be required (i.e. abdominal pain, nausea and/or vomiting), supported by the finding of elevated serum amylase greater than 300 U/L. For the radiological diagnosis, if applicable, computerised tomography (CT) and/or ultrasound scan (USS) evidence of acute pancreatitis will be accepted.

Exclusion criteria

i) Patients under 16 years of age.
ii) Prisoners.
iii) Any patient that lacks capacity to consent.

The additional two exclusions below apply only to those patients being considered for the nested cohort study:

iv) Patients not able to undergo MRI scanning for technical reasons will be excluded (e.g. those with cochlear implants, implanted pacemaker)
v) Patients with a known allergy to salbutamol

Co-enrolment to other research studies, including drug, interventional and long-term follow-up studies, will be permitted if this has been agreed and documented by the Chief Investigators of co-enrolling studies.

Statistical analysis plan

Full details of the statistical analysis will be documented in the statistical analysis plan developed prior to any analysis. Any deviations from this will be documented. We will present the data descriptively, where data is categorical numbers and percentages will be presented and where data is continuous number, mean, standard deviation, median, 25th centile, 75th centile, min and max will be presented. Annual incidence of type 3c diabetes will be given as a percentage with an accompanying 95% confidence interval. For the nested cohort we will present the change in fibrosis index over time graphically with accompanying descriptive statistics, and where appropriate include details of the index episode including

For peer review only - http://bmjopen.bmj.com/site/about/guidelines.xhtml

aetiology, severity and pre-existing medical background as covariates wherever possible. Quality of assessment will be included as a potential confounder.

Sample size calculation

Overall sample size calculation has been performed to illustrate how well we will be able to estimate the annual incidence of type 3c diabetes in patients with acute pancreatitis with varying sample sizes[26 27]. The incidence of type 3c DM was chosen for the purposes of establishing a prospective cohort size because it is specific, measurable, clinically relevant and offers a practice-changing opportunity for intervention. With a sample of 323 we would have 80% power to be able to show a difference from the incidence of diabetes mellitus in the general population (6%) if we observed an incidence in the cohort of 10% or greater. Moreover, this sample size will support the evaluation of the secondary endpoints, allowing us to adjust for any confounding factors.

Nested cohort sample size has been calculated to detect the difference in the 3-months to 36-month change in pancreatic fibrosis index between those with AP with MODS and those with AP without MODS based on Banerjee et al. estimates of fibrosis index using multiparameter MRI[28]. Based on a sample size of 20 participants per group, using a two-sided two-sample test with a 5% level of significance and 80% power, we would be able to detect an effect size of 0.909 (where the effect size is the absolute difference in means divided by common standard deviation). To allow for potential dropouts in this part of the study (death, withdrawal) a minimum sample size of 25 individuals will be recruited for the nested cohort.

Data management

Data will be collected either directly onto an electronic case report form on REDCap (Research Electronic Data Capture) tools hosted at University of Edinburgh[29 30] or onto paper data collection sheets which will be entered onto the electronic form at site. All participants will be allocated a unique study number which will be used in all electronic and paper data collection tools. The study link-anonymised data will be stored on secure password-protected, regularly backed-up, computerised storage in accordance to secure data protection principles of the University of Edinburgh/CRF at study sites.

For peer review only - http://bmjopen.bmj.com/site/about/guidelines.xhtml

Personal contact details and the CHI (Community Health Index) number are required for follow-up and tracking electronic health records, and will initially be kept on paper at participating sites in a secure storage area with restricted access. These details will ultimately be stored on an encrypted database within NHS Lothian. Personal contact details and CHI numbers will not be entered onto the study database.

The Chief Investigator (DJM) will have access to the data during all stages of the study. The analysis will be performed in secure premises at the University of Edinburgh and NHS Lothian by the Chief Investigator and the assigned statistician. The Chief Investigator will have control of and act as the custodian for the data generated by the study.

Participants

Consent

Potential study participants will be provided with a written information sheet and a written consent form. In addition, participants will meet with a member of the clinical research facility (CRF) team to receive a full explanation of the nature and purpose of the study, with the opportunity to ask questions. It will be made clear that the individual may withdraw from the study at any time without giving a reason and with no consequence to their current or future care. Recruitment and informed consent will be performed by the investigator/member of the CRF team at the participating site. If individuals wish to participate, they will sign a consent form, countersigned by the investigator/CRF researcher. In addition, consent will be obtained to inform the participants' GP and Consultant in charge of their care.

Patient decision period

Potential participants who have been invited to participate will be given a period of up to 2 days (minimum 1 hour) to read the relevant information sheets, to contact a member of the study team to get further information and ask questions, before they are invited to participate in a conversation and commence the process of fully-informed consent.

Withdrawal of participants

Participants will be withdrawn from the clinical research study in the following circumstances:

i) In cases of withdrawal of informed consent, where capacity exists.
ii) In cases of withdrawal of consent by the participant's representative for adults with incapacity.

For peer review only

Participants who wish to withdraw from the study will be given the option to permit ongoing use of data and samples that have already been collected, and/or future recording and usage of routinely collected clinical data and results. This will be clearly documented on the patient consent form. Patients that become uncontactable will have any previously collected data included in the study unless consent is withdrawn as above.

Benefits to participants

No direct benefit is expected for the participants. We envisage that the results of this study will benefit other patients with AP in the future, and society as a whole by advancing scientific knowledge on this condition. We believe that this study may lead to new strategies in the treatment of AP which will prevent or ameliorate the impact of organ dysfunction. This will help to maximise the health, and life expectancy, of patients with AP.

Potential burden to participants

The participants will be subjected to peripheral venous blood sampling which may cause discomfort or pain. In order to minimise these effects, blood sampling will be performed by appropriately trained and experienced members of the research team, in adherence to the local guidelines for blood sampling. Where possible, and only if the specific study venepuncture time-points coincide, clinical care samples will be taken at the same time to reduce the number of venepunctures that any one participant sustains.

The collection of urine and stool samples is not expected to cause any harm or discomfort; however, some minimal inconvenience may be caused. In order to address this, a convenient time for the participants and the clinical care team will be chosen, and a full explanation of the purpose of these tests will be provided. The participants' dignity and privacy will be respected at all times.

The six-minute walk-test is not anticipated to cause any harm or discomfort to participants. Pulse wave analysis is not expected to cause any significant adverse effects. Inhaled salbutamol may cause a very slight peripheral tremor, but no other expected harm or discomfort.

CPEX testing is associated with a very low risk of serious complications; however, this is related to the severity of co-morbid disease such as coronary artery disease (CAD)[25]. For those

For peer review only

participants with identified CAD, the opinion of a cardiologist will be sought on the suitability of the participant for this test.

Assessment of pulmonary function using spirometry is not known to be associated with any significant adverse events[31]. Participants may experience some discomfort or dizziness during the test due to the requirement for complete exhalation[32]. Spirometry is associated with increased myocardial strain, and a rise in intrathoracic, intraabdominal and intraocular pressures, and consequently will not be performed on participants with the following: recent head/neck, thoracic or abdominal surgery, recent myocardial infarction, retinal detachment, resting pulse greater than 120bpm, pregnant, severe heart failure or arrhythmia, pneumothorax, aortic or cerebral aneurysm[33].

MRI scans are not expected to pose risks to the majority of participants, in the absence of known contraindications. Participants will be assessed prior to the scan for any potential contraindications to the procedure such as mechanical heart valves, pacemakers, metallic prostheses and cochlear implants[34].

ETHICS AND DISSEMINATION

This study has been reviewed as IRAS Number 178615 by the South-East Scotland Research Ethics Committee under the application numbers (REC Number) 16/SS/0065 and has been given a favourable opinion. NHS Scotland R&D approval have been granted. The study sponsor is NHS Lothian/University of Edinburgh ACCORD. The trial is registered at ClinicalTrials.gov (NCT03342716).

Results of the study will be presented at local, national and international medical meetings. The findings of the study will be published in peer-reviewed medical/scientific journals and made open-access on acceptance. If appropriate, the results of the study will be disseminated via press releases by the University of Edinburgh. Information may also be disseminated to the general public via public engagement and community outreach programmes.

Patient and Public Involvement statement: Patients who have had an episode of AP and who participate in the APPLe (acute pancreatitis patient liaison group) were invited to input and contributed to the concepts and the trial design.

For peer review only

VERSIONING: This paper is based on the full protocol V11, dated 23rd September 2019

AUTHOR CONTRIBUTIONS

DJM had the idea and wrote the funding application with statistics input from CG. DJM, CV, CG and JB developed and drafted the clinical study protocol and ethics application. XZ, AW, DC, SS and DM designed and contributed additional details of the investigations. MS and AW designed and built the data collection system. DJM, JB, MG, KW, Audrey White, LM, EC, ET, AS, DD, RM, SC and other RESORP team members initiated the project. AS, RM, DJM drafted the manuscript. All authors contributed to and approved the final version of the manuscript.

ROLE OF SPONSOR AND FUNDER

The sponsor and funder has no direct role or influence in the design or conduct of the study, or the decision to submit the protocol for publication.

FUNDING

This study is funded by the United Kingdom Medical Research Council through a Senior Clinical Fellowship to Damian J. Mole. Ref: MR/P008887/1.

DECLARATION OF INTERESTS

No individual has an actual or perceived conflict of interest with this study. DJM wishes to declare that he holds a senior position in Kynos Therapeutics Ltd, a private company developing medicines for systemic inflammation and cancer, and that he has previously received funding for collaborative studies on AP from GSK.

For peer review only

REFERENCES

1. Yadav D, Lowenfels AB. The epidemiology of pancreatitis and pancreatic cancer. *Gastroenterology* 2013;144(6):1252-61. doi: 10.1053/j.gastro.2013.01.068 [published Online First: 2013/04/30]

2. Banks PA. Epidemiology, natural history, and predictors of disease outcome in acute and chronic pancreatitis. *Gastrointest Endosc* 2002;56(6 Suppl):S226-30. doi: 10.1067/mge.2002.129022 [published Online First: 2002/11/26]

3. Lankisch PG, Apte M, Banks PA. Acute pancreatitis. *Lancet* 2015;386(9988):85-96. doi: 10.1016/S0140-6736(14)60649-8 [published Online First: 2015/01/27]

4. McKay CJ, Evans S, Sinclair M, et al. High early mortality rate from acute pancreatitis in Scotland, 1984-1995. *Br J Surg* 1999;86(10):1302-5. doi: 10.1046/j.1365-2168.1999.01246.x [published Online First: 1999/10/30]

5. Roberts SE, Williams JG, Meddings D, et al. Incidence and case fatality for acute pancreatitis in England: geographical variation, social deprivation, alcohol consumption and aetiology--a record linkage study. *Aliment Pharmacol Ther* 2008;28(7):931-41. doi: 10.1111/j.1365-2036.2008.03809.x [published Online First: 2008/07/24]

6. Skouras C, Hayes AJ, Williams L, et al. Early organ dysfunction affects long-term survival in acute pancreatitis patients. *HPB (Oxford)* 2014;16(9):789-96. doi: 10.1111/hpb.12259 [published Online First: 2014/04/10]

7. Mole DJ, Gungabissoon U, Johnston P, et al. Identifying risk factors for progression to critical care admission and death among individuals with acute pancreatitis: a record linkage analysis of Scottish healthcare databases. *BMJ open* 2016;6(6):e011474. doi: 10.1136/bmjopen-2016-011474 [published Online First: 2016/06/18]

8. Dellinger EP, Forsmark CE, Layer P, et al. Determinant-based classification of acute pancreatitis severity: an international multidisciplinary consultation. *Ann Surg* 2012;256(6):875-80. doi: 10.1097/SLA.0b013e318256f778 [published Online First: 2012/06/28]

9. Banks PA, Bollen TL, Dervenis C, et al. Classification of acute pancreatitis--2012: revision of the Atlanta classification and definitions by international consensus. *Gut*

For peer review only

For peer review only - http://bmjopen.bmj.com/site/about/guidelines.xhtml

2013;62(1):102-11. doi: 10.1136/gutjnl-2012-302779 [published Online First: 2012/10/27]

10. Johnson CD, Abu-Hilal M. Persistent organ failure during the first week as a marker of fatal outcome in acute pancreatitis. *Gut* 2004;53(9):1340-4. doi: 10.1136/gut.2004.039883 [published Online First: 2004/08/13]

11. Garg PK, Singh VP. Organ Failure Due to Systemic Injury in Acute Pancreatitis. *Gastroenterology* 2019;156(7):2008-23. doi: 10.1053/j.gastro.2018.12.041 [published Online First: 2019/02/16]

12. al. Ne. Molecular patterns in acute pancreatitis reflect generalisable endotypes of the host response to systemic injury in humans., 2020.

13. Skouras C, Hayes A, Williams I. Early organ dysfunction affects long term survival in acute pancreatitis patients. *HPB* 2013;16(9):789-96.

14. Nøjgaard C. Prognosis of acute and chronic pancreatitis: a 30 year follow up of a Danish cohort. *Dan Med Bull* 2010;57(12):B4228.

15. Lankisch P, Breuer N, Bruns A. Natural history of acute pancreatitis: a long-term population-based study. *Am J Gastroenterol* 2009;104(11):2797-805.

16. Cavestro G, Leandro G, Di Leo M. A single-centre propsective, cohort study of the natural history of acute pancreatitis. *Dig Liver Dis* 2015;47(3):205-10.

17. Appelros S, Lingren S, Borgstrom A. Short and long-term outcome of severe acute pancreatitis. *Eur J Surg* 2001;167(4):281-86.

18. Halonen K, Pettila V, Leppaniemi A. Long-term health-related quality of life in survivors of severe acute pancreatitis. *Intensive Care Med* 2003;29(5):782-86.

19. Ciesla N, Dinglas V, Fan E, et al. Manual Muscle Testing: A Method of Measuring Extremity Muscle Strength Applied to Critically Ill Patients. *JoVE* 2011(50):e2632. doi: doi:10.3791/2632

20. Sway balance 2020 [cited 2020 20 February]. Available from: http://swaymedical.com/system/balance.

21. Amick RZ, Chaparro A, Patterson JA. Test-Retest Reliability of the sway Balance Mobile Application. *Journal of Mobile Technology in Medicine* 2015;4(2):40-47. doi: 10.7309/jmtm.4.2.6

22. Six-minute walk test 2020 [cited 2020 20 February]. Available from: http://www.pulmonaryrehab.com.au/index.asp?page=19.

For peer review only

For peer review only - http://bmjopen.bmj.com/site/about/guidelines.xhtml

23. Guidelines for the measurement of respiratory function. Recommendations of the British Thoracic Society and the Association of Respiratory Technicians and Physiologists. *Respir Med* 1994;88(3):165-94. [published Online First: 1994/03/01]

24. K W, JE H, DY S, et al. Principles of Exercise Testing and Interpretation. Philadelphia, PA: Lippincott Williams & Wilkins 2005:133-156.

25. American Thoracic S, American College of Chest P. ATS/ACCP Statement on cardiopulmonary exercise testing. *Am J Respir Crit Care Med* 2003;167(2):211-77. doi: 10.1164/rccm.167.2.211 [published Online First: 2003/01/14]

26. Ewald N, Kaufmann C, Raspe A, et al. Prevalence of diabetes mellitus secondary to pancreatic diseases (type 3c). *Diabetes Metab Res Rev* 2012;28(4):338-42. doi: 10.1002/dmrr.2260 [published Online First: 2011/11/29]

27. Woodmansey C, McGovern AP, McCullough KA, et al. Incidence, Demographics, and Clinical Characteristics of Diabetes of the Exocrine Pancreas (Type 3c): A Retrospective Cohort Study. *Diabetes Care* 2017;40(11):1486-93. doi: 10.2337/dc17-0542 [published Online First: 2017/09/02]

28. Banerjee R, Pavlides M, Tunnicliffe EM, et al. Multiparametric magnetic resonance for the non-invasive diagnosis of liver disease. *J Hepatol* 2014;60(1):69-77. doi: 10.1016/j.jhep.2013.09.002 [published Online First: 2013/09/17]

29. Harris PA, Taylor R, Minor BL, et al. The REDCap consortium: Building an international community of software platform partners. *J Biomed Inform* 2019;95:103208. doi: 10.1016/j.jbi.2019.103208 [published Online First: 2019/05/13]

30. Harris PA, Taylor R, Thielke R, et al. Research electronic data capture (REDCap)--a metadata-driven methodology and workflow process for providing translational research informatics support. *J Biomed Inform* 2009;42(2):377-81. doi: 10.1016/j.jbi.2008.08.010 [published Online First: 2008/10/22]

31. Lin K, Watkins B, Johnson T, et al. Screening for chronic obstructive pulmonary disease using spirometry: summary of the evidence for the U.S. Preventive Services Task Force. *Ann Intern Med* 2008;148(7):535-43. doi: 10.7326/0003-4819-148-7-200804010-00213 [published Online First: 2008/03/05]

32. Buist AS, Vollmer WM, Sullivan SD, et al. The Burden of Obstructive Lung Disease Initiative (BOLD): rationale and design. *COPD* 2005;2(2):277-83. [published Online First: 2006/12/02]

For peer review only

33. Coates AL, Graham BL, McFadden RG, et al. Spirometry in primary care. *Can Respir J* 2013;20(1):13-21. doi: 10.1155/2013/615281 [published Online First: 2013/03/05]

34. Dill T. Contraindications to magnetic resonance imaging: non-invasive imaging. *Heart* 2008;94(7):943-8. doi: 10.1136/hrt.2007.125039 [published Online First: 2008/06/17]

For peer review only

SPIRIT

STANDARD PROTOCOL ITEMS: RECOMMENDATIONS FOR INTERVENTIONAL TRIALS

Study Protocol for RESORP – Resolution of Organ Injury in Acute Pancreatitis – an observational prospective cohort study

SPIRIT 2013 Checklist: Recommended items to address in a clinical trial protocol and related documents*

Section/item	Item No	Description
**Administrative information**		
Title	1	Descriptive title identifying the study design, population, interventions, and, if applicable, trial acronym – yes, line 1
Trial registration	2a	Trial identifier and registry name. If not yet registered, name of intended registry – yes, line 53
	2b	All items from the World Health Organization Trial Registration Data Set
Protocol version	3	Date and version identifier – yes, line 431
Funding	4	Sources and types of financial, material, and other support – declared, line 445
Roles and responsibilities	5a	Names, affiliations, and roles of protocol contributors – yes, p1 and p16
	5b	Name and contact information for the trial sponsor – yes, line 419
	5c	Role of study sponsor and funders, if any, in study design; collection, management, analysis, and interpretation of data; writing of the report; and the decision to submit the report for publication, including whether they will have ultimate authority over any of these activities – yes, 441
	5d	Composition, roles, and responsibilities of the coordinating centre, steering committee, endpoint adjudication committee, data management team, and other individuals or groups overseeing the trial, if applicable (see Item 21a for data monitoring committee) – not applicable
**Introduction**		
Background and rationale	6a	Description of research question and justification for undertaking the trial, including summary of relevant studies (published and unpublished) examining benefits and harms for each intervention – yes, p4
	6b	Explanation for choice of comparators – not applicable
Objectives	7	Specific objectives or hypotheses – clearly stated, line 100

For peer review only

Trial design	8	Description of trial design including type of trial (eg, parallel group, crossover, factorial, single group), allocation ratio, and framework (eg, superiority, equivalence, noninferiority, exploratory) – line 126

Methods: Participants, interventions, and outcomes

Study setting	9	Description of study settings (eg, community clinic, academic hospital) and list of countries where data will be collected. Reference to where list of study sites can be obtained – clearly stated, line 122
Eligibility criteria	10	Inclusion and exclusion criteria for participants. If applicable, eligibility criteria for study centres and individuals who will perform the interventions (eg, surgeons, psychotherapists) – clearly stated, p10 and 11
Interventions	11a	Interventions for each group with sufficient detail to allow replication, including how and when they will be administered – not applicable
	11b	Criteria for discontinuing or modifying allocated interventions for a given trial participant (eg, drug dose change in response to harms, participant request, or improving/worsening disease) – not applicable
	11c	Strategies to improve adherence to intervention protocols, and any procedures for monitoring adherence (eg, drug tablet return, laboratory tests) – not applicable
	11d	Relevant concomitant care and interventions that are permitted or prohibited during the trial– not applicable
Outcomes	12	Primary, secondary, and other outcomes, including the specific measurement variable (eg, systolic blood pressure), analysis metric (eg, change from baseline, final value, time to event), method of aggregation (eg, median, proportion), and time point for each outcome. Explanation of the clinical relevance of chosen efficacy and harm outcomes is strongly recommended– clearly stated, p6
Participant timeline	13	Time schedule of enrolment, interventions (including any run-ins and washouts), assessments, and visits for participants. A schematic diagram is highly recommended (see Figure) – clearly stated, line 136
Sample size	14	Estimated number of participants needed to achieve study objectives and how it was determined, including clinical and statistical assumptions supporting any sample size calculations– clearly stated, line 314
Recruitment	15	Strategies for achieving adequate participant enrolment to reach target sample size– clearly stated p10

Methods: Assignment of interventions (for controlled trials)

Allocation:

Sequence generation	16a	Method of generating the allocation sequence (eg, computer-generated random numbers), and list of any factors for stratification. To reduce predictability of a random sequence, details of any planned restriction (eg, blocking) should be provided in a separate document that is unavailable to those who enrol participants or assign interventions – not applicable
Allocation concealment mechanism	16b	Mechanism of implementing the allocation sequence (eg, central telephone; sequentially numbered, opaque, sealed envelopes), describing any steps to conceal the sequence until interventions are assigned – not applicable
Implementation	16c	Who will generate the allocation sequence, who will enrol participants, and who will assign participants to interventions – not applicable
Blinding (masking)	17a	Who will be blinded after assignment to interventions (eg, trial participants, care providers, outcome assessors, data analysts), and how – not applicable
	17b	If blinded, circumstances under which unblinding is permissible, and procedure for revealing a participant's allocated intervention during the trial – not applicable
**Methods: Data collection, management, and analysis**		
Data collection methods	18a	Plans for assessment and collection of outcome, baseline, and other trial data, including any related processes to promote data quality (eg, duplicate measurements, training of assessors) and a description of study instruments (eg, questionnaires, laboratory tests) along with their reliability and validity, if known. Reference to where data collection forms can be found, if not in the protocol – clearly stated. Line 166
	18b	Plans to promote participant retention and complete follow-up, including list of any outcome data to be collected for participants who discontinue or deviate from intervention protocols – clearly stated, line 317
Data management	19	Plans for data entry, coding, security, and storage, including any related processes to promote data quality (eg, double data entry; range checks for data values). Reference to where details of data management procedures can be found, if not in the protocol – clearly stated, line 333
Statistical methods	20a	Statistical methods for analysing primary and secondary outcomes. Reference to where other details of the statistical analysis plan can be found, if not in the protocol – clearly stated, p11
	20b	Methods for any additional analyses (eg, subgroup and adjusted analyses) – clearly stated, p11

	20c	Definition of analysis population relating to protocol non-adherence (eg, as randomised analysis), and any statistical methods to handle missing data (eg, multiple imputation) – clearly stated, p11
**Methods: Monitoring**		
Data monitoring	21a	Composition of data monitoring committee (DMC); summary of its role and reporting structure; statement of whether it is independent from the sponsor and competing interests; and reference to where further details about its charter can be found, if not in the protocol. Alternatively, an explanation of why a DMC is not needed – not applicable
	21b	Description of any interim analyses and stopping guidelines, including who will have access to these interim results and make the final decision to terminate the trial – not applicable
Harms	22	Plans for collecting, assessing, reporting, and managing solicited and spontaneously reported adverse events and other unintended effects of trial interventions or trial conduct – clearly stated, p14
Auditing	23	Frequency and procedures for auditing trial conduct, if any, and whether the process will be independent from investigators and the sponsor– not applicable
**Ethics and dissemination**		
Research ethics approval	24	Plans for seeking research ethics committee/institutional review board (REC/IRB) approval– obtained, line 416
Protocol amendments	25	Plans for communicating important protocol modifications (eg, changes to eligibility criteria, outcomes, analyses) to relevant parties (eg, investigators, REC/IRBs, trial participants, trial registries, journals, regulators) – clearly stated, line 421
Consent or assent	26a	Who will obtain informed consent or assent from potential trial participants or authorised surrogates, and how (see Item 32) – clearly stated, line 351
	26b	Additional consent provisions for collection and use of participant data and biological specimens in ancillary studies, if applicable– clearly stated, line 371
Confidentiality	27	How personal information about potential and enrolled participants will be collected, shared, and maintained in order to protect confidentiality before, during, and after the trial– clearly stated, line 332
Declaration of interests	28	Financial and other competing interests for principal investigators for the overall trial and each study site– clearly stated, line 448

review only

Access to data	29	Statement of who will have access to the final trial dataset, and disclosure of contractual agreements that limit such access for investigators– clearly stated, line 345
Ancillary and post-trial care	30	Provisions, if any, for ancillary and post-trial care, and for compensation to those who suffer harm from trial participation – not applicable
Dissemination policy	31a	Plans for investigators and sponsor to communicate trial results to participants, healthcare professionals, the public, and other relevant groups (eg, via publication, reporting in results databases, or other data sharing arrangements), including any publication restrictions – clearly stated, line 421
	31b	Authorship eligibility guidelines and any intended use of professional writers– not applicable
	31c	Plans, if any, for granting public access to the full protocol, participant-level dataset, and statistical code– not applicable yet.
**Appendices**		
Informed consent materials	32	Model consent form and other related documentation given to participants and authorised surrogates – not included
Biological specimens	33	Plans for collection, laboratory evaluation, and storage of biological specimens for genetic or molecular analysis in the current trial and for future use in ancillary studies, if applicable– clearly stated, line 154 and following

*It is strongly recommended that this checklist be read in conjunction with the SPIRIT 2013 Explanation & Elaboration for important clarification on the items. Amendments to the protocol should be tracked and dated. The SPIRIT checklist is copyrighted by the SPIRIT Group under the Creative Commons "Attribution-NonCommercial-NoDerivs 3.0 Unported" license.

For peer review only - http://bmjopen.bmj.com/site/about/guidelines.xhtml
